# The Opening of Our Medical Schools

**Published:** 1857-12

**Authors:** 


*The opening of our Medical Schools.*—However far we may be removed from the influence of medical schools, we must always feel an interest in the educational progress of our profession, and we cannot but look back with respect and veneration upon the college which sent us out to minister to the ills and sufferings of humanity, and watch with peculiar interest the progress of the institution of which we have such cheerful remembrances. With some of our readers, the time which is to be passed over in our retrospective musings is a lifetime in itself; years of toil and hardship, filled with the recollection of the painful scenes, the peculiar annoyances and mortifications which fall to the lot of every practitioner, but at the same time studded with bright spots, the enviable satisfaction which all must experience in having done good; and sometimes smoothed by the grateful consideration and respect which our office among men occasionally awakens. It is impossible to pass over this period, and to look back on our old college days with other feelings than those of pleasure and satisfaction. Some have realized the fond hopes and aspirations which were called out by the contemplation of the deeds of great men in our ranks, and the study of the character of eminent teachers of our own country, with whom it has been their good fortune to hold the intimate relation of pupil and instructor; while all have become useful members of society, and if they have not made their light shine before all men, they have the consciousness of having done at least their portion of good, no matter how limited the sphere to which their labors have been confined.

In spite of the hard times, the ranks of the profession have nowhere been very much thinned, and in some of our larger institutions, have been somewhat swelled by the access of medical students. The Jefferson school of Philadelphia, which for some years has stood at the head of the list as regards numbers, has even a larger class the present session than it had the year before; the venerable University of Penn. has, we believe, a good class, though falling somewhat short of last year. About the remaining Philadelphia schools we have not heard any definite report, but we conclude that they are in a fair way. The New York colleges are somewhat thinner in classes than they were a year ago, but still are doing well, and the able corps of medical teachers which has congregated in the great metropolis, is hard at work in the schools and hospitals, helping the student to avail himself of the advantages which that great city has at its disposal. The calamity which overtook the Louisville University, has made no-break in its order of teaching, the faculty having already replaced the old edifice which was destroyed by fire last year, by a new one of even greater convenience.

Judging of the state of the smaller schools by the Buffalo Medical College, they are not behind the past year in prosperity. The Buffalo school is unfortunately situated for large classes; a great many of the students who properly belong here, disappear into the capacious maw of Philadelphia, or are tempted by the inducements which are presented by a great city like New York. On the other side, we have the free school of Ann Arbor, which diminishes somewhat the number, but which we conceive rather winnows from us the chaff, (though the chaff's money is as good as any other,) but leaves us still a good class of intelligent and earnest men. The Buffalo Medical College has always preserved its integrity; the fees for lectures have not been diminished, and with its able and zealous faculty will at all times command a good class. The number of students will probably be about the same as last year; the term opened as usual on the first of November, under favorable auspices, and the hospital clinic has thus far been unusually replete with interesting medical and surgical cases, some of which have been exceedingly rare and instructive. The introductory was delivered by Prof. Flint, who presented some of the favorable aspects of the profession of Medicine; it was an able and interesting discourse, and was listened to with marked attention by the class and quite a number of citizens. F.

